# The perioperative management of cesarean section in a patient with FXIII deficiency and placenta previa: a case report

**DOI:** 10.1186/s40981-022-00563-y

**Published:** 2022-09-15

**Authors:** Kei Ueda, Kentaro Miyoshi, Shinichi Kai

**Affiliations:** 1 Department of Anesthesia, Kishiwada City Hospital, Osaka, Japan; 2 Department of Anesthesia, Kyoto University Hospital, Kyoto, Japan

To the editor, Factor XIII (FXIII), called fibrin stabilizing factor, plays a role in uterine hemostasis and placental maintenance. FXIII deficiency is a risk factor for miscarriage and supplementation is helpful for continued pregnancy [1], whereas the perioperative management of cesarean section (CS) in pregnant patients with FXIII deficiency is unclear. Here, we report a case of the perioperative management of CS in a patient with FXIII deficiency and placenta previa.

A 38-year-old woman was admitted to Kyoto University Hospital at 17 weeks gestation because a subchorionic hematoma was detected on ultrasound examination. Although her platelet count, Prothrombin Time-International Normalized Ratio, and APTT were within the normal range, subchorionic hematoma was increased. Subsequently examined coagulation factors revealed low FXIII activity level (59%), leading to the diagnosis of FXIII deficiency. The FXIII level was maintained above 70% with the administration of FXIII concentrates (10 U/kg) to maintain pregnancy. Elective CS at 37 weeks of gestation was performed. Her FXIII level was maintained within the normal range (75%), and then we performed spinal anesthesia. After delivery of the baby, the placenta thought to be placenta previa turned out to be placenta accreta, and it was difficult to deliver. The amount of blood loss increased (4700 ml). Consequently, we switched to general anesthesia and transfused 2 U of red blood cells, 2 U of fresh frozen plasma, and 800 ml of autologous blood. Furthermore, we also administered 480 U of FXIII concentrates. When she was transferred to the ICU, her FXIII level was 87%. She was discharged on postoperative day 32 with no major complications.

There are currently no guidelines for the use of neuraxial anesthesia in patients with FXIII deficiency during CS. In some case reports, neuraxial anesthesia was safely performed with careful management of FXIII levels [2, 3]. We performed spinal anesthesia because the FXIII level was maintained within the normal range. We administered FXIII concentrates (10 U/kg) in addition to allogeneic transfusion to prevent a decrease in FXIII levels after bleeding. Some have recommended that an additional dose of FXIII should be administered during labor and delivery for safet y[2, 4]. Korte et al. also demonstrated that early intraoperative FXIII substitution would allow a decrease in the loss of clot firmness [5].

Potential thrombogenicity of the procoagulant factors used in clinical practice must be a concern; however, factor XIII supplementation has thus far not been linked to an increased risk of thromboembolism and we have demonstrated that FXIII can be safely used in the perioperative period in pregnant women at high risk of embolism. Therefore, we suggested that additional FXIII concentrates be administered in these patients during CS without hesitation, especially when massive bleeding occurs.

We demonstrated successful perioperative management in a patient with FXIII deficiency, although massive bleeding occurred. Spinal anesthesia could be performed if FXIII levels are carefully managed. When massive bleeding occurs, additional FXIII concentrates could be administered and FXIII levels and postpartum hemorrhage should be evaluated carefully.

## Data Availability

Not applicable
